# Massive Pericardial Effusion and Type 3 Cardiorenal Syndrome as the Inaugural Presentation of Late-Onset Systemic Lupus Erythematosus in a Septuagenarian

**DOI:** 10.7759/cureus.102066

**Published:** 2026-01-22

**Authors:** Sergio David Angulo, Cesar Cortes, Manuela Orozco, Juan Diego Trujillo Loaiza, Diego Ruiz, Juan Pablo Rodríguez, Laura Fernanda Giraldo Nieto

**Affiliations:** 1 Teaching and Research Unit, Caja de Compensación Familiar de Risaralda - Salud Comfamiliar, Pereira, COL; 2 Internal Medicine, Universidad de Manizales, Manizales, COL; 3 Orthopedic Surgery, Universidad de Manizales, Manizales, COL; 4 Ophthalmology, Universidad de Manizales, Manizales, COL; 5 Internal Medicine, Hospital San Juan de Dios Riosucio, Riosucio, COL; 6 Obstetrics and Gynecology, Hospital San Juan de Dios Riosucio, Riosucio, COL

**Keywords:** chronic kidney disease, heart failure, late-onset systemic lupus erythematosus, lupus, lupus nephropathy

## Abstract

Systemic lupus erythematosus (SLE) is a chronic autoimmune disease characterized by inflammation and immune-mediated damage with mucocutaneous, musculoskeletal, cardiopulmonary, and renal involvement. It typically affects younger women; however, late-onset presentations can occur and often display distinct clinical features. We report the case of a septuagenarian woman who presented with massive pericardial effusion and type 3 cardiorenal syndrome. Further evaluation revealed class IV lupus nephritis, confirming a diagnosis of late-onset SLE according to the 2019 European League Against Rheumatism (EULAR)/American College of Rheumatology (ACR) criteria. Cardiac and serous membrane involvement is more common in late-onset SLE than in classical SLE.

## Introduction

Systemic lupus erythematosus (SLE) is a chronic autoimmune disease characterized by inflammation and immune-mediated damage with mucocutaneous, musculoskeletal, cardiopulmonary, and renal involvement [1]. It predominantly affects females, with a peak presentation between the second and fifth decades of life [2]. Diagnosis is based on the 2019 European League Against Rheumatism (EULAR)/American College of Rheumatology (ACR) criteria, which encompass clinical and immunological domains [3].

Late-onset SLE, defined as disease onset at or after 50 years of age [4], presents distinct challenges. The prevalence of late-onset SLE varies widely, ranging from 9% in Spain to as low as 1% in cohorts of patients aged 60 years or older in Japan. Clinically, late-onset SLE is often characterized by a higher frequency of serositis, lung disease, and cardiac involvement. At the same time, malar rash, nephritis, and neuropsychiatric manifestations are less common than in early-onset disease. Despite typically lower disease activity, older patients often face a poorer prognosis due to irreversible organ damage and delayed diagnosis. We present a clinical case of an elderly patient with late-onset SLE who presented with massive pericardial effusion and cardiorenal syndrome (CRS), highlighting the complexity of this diagnosis in the geriatric population.

## Case presentation

**Figure 1:**
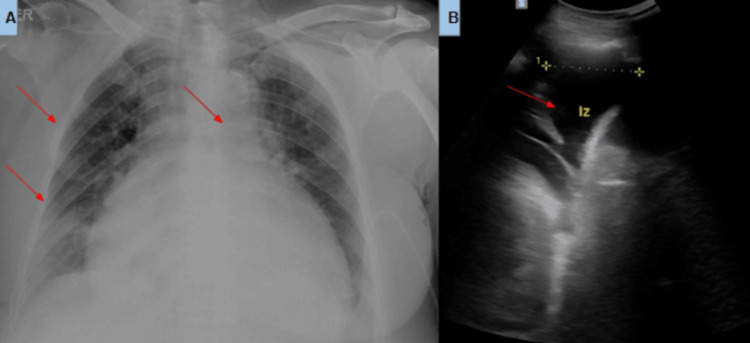
(A) Anteroposterior chest X-ray demonstrating cotton-wool infiltrates (arrows), cardiomegaly, and bilateral pericardial and pleural effusions. (B) Lung ultrasound image showing a large, 1000 mL left pleural effusion (arrow)

A 76-year-old woman with a history of hypertension was referred to the emergency department due to a three-month history of progressive dyspnea (New York Heart Association (NYHA) functional class IV), asthenia, and adynamia [5]. Upon admission, she presented with hemodynamic instability (blood pressure 155/102), hypoxemia (saturation 89%), and anasarca. Transthoracic echocardiogram revealed a reduced left ventricular ejection fraction (LVEF) of 39% with global hypokinesis. A massive circumferential pericardial effusion (28 mm) was noted, exhibiting the "swinging heart" phenomenon. Significant signs of cardiac pre-tamponade were present, including diastolic collapse of the right atrium, dilated inferior vena cava without inspiratory collapse, and marked respiratory variation in transmitral (>30%) and transtricuspid (>25%) flows. Chest radiography demonstrated cardiomegaly, bilateral cotton-wool infiltrates, and bilateral pleural effusions. She was transferred to the intensive care unit for vasoactive support and underwent ultrasound-guided pericardiocentesis, draining 800 mL of transudative fluid (Figure 1).

Following clinical stabilization, the patient was transferred to the general ward; however, generalized edema persisted. The Nephrology service was consulted to broaden the diagnostic evaluation. Subsequent laboratory tests revealed elevated NT-proBNP, azotemia, nephrotic-range proteinuria, hypoalbuminemia, hypocomplementemia, and positive antinuclear antibodies (ANAs) (Table 1).

**Table 1:** Laboratory data

Parameter	Patient value	Reference range
24-hour urine protein (mg/24h)	5472	0-0
Albumin (g/dL)	1.87	3.5-5.5
Brain natriuretic peptide (pg/mL)	21000	0-450
Creatinine (mg/dL)	4.0	0.7-1.3
Blood urea nitrogen (BUN) (mg/dL)	40	6-20
Erythrocyte sedimentation rate (ESR) (mm/h)	45	0-20
Complement C3 (mg/dL)	50	83-193
Complement C4 (mg/dL)	5	15-57
Antinuclear antibody (ANA) titer	1:640 (positive)	>1:80
Double-stranded DNA (dsDNA)	Negative	Negative
Extractable nuclear antigens (ENAs)	Negative	Negative
SS-A (Ro) antibody	Negative	Negative
SS-B (La) antibody	Negative	Negative
Anti-RNP antibody	Negative	Negative
Lupus anticoagulant (LA)	Negative	Negative
Anti-topoisomerase I (Scl-70) antibody	Scleroderma pattern associated	Negative

**Figure 2:**
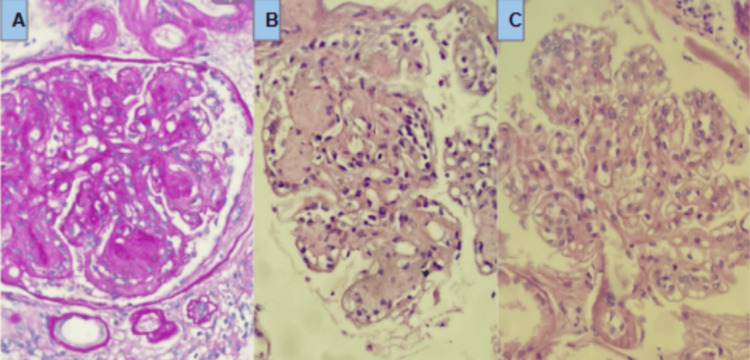
Renal biopsy (40×). Sections stained with (A) hematoxylin and eosin (H&E), (B) trichrome, and (C) silver stain demonstrating features of membranoproliferative glomerulonephritis

A renal ultrasound demonstrated signs of bilateral diffuse chronic nephropathy with cortical atrophy. A renal biopsy confirmed immune complex-mediated glomerulonephritis compatible with class IV lupus nephritis (Figure 2). Based on these findings, the patient met the 2019 EULAR/ACR criteria with a total score of 18 (lupus nephritis, low complement, and pericardial effusion involvement).

Given the patient's age and massive effusion, malignancy was a potential differential diagnosis. However, the initial clinical picture was dominated by severe cardiac failure with significantly elevated natriuretic peptides, directing the immediate focus toward hemodynamic management. The subsequent rapid identification of nephrotic syndrome and autoimmunity strongly pointed towards a systemic autoimmune etiology, making a primary neoplastic or infectious cause unlikely without the need for exhaustive workup.

Immunosuppressive therapy was initiated with methylprednisolone (500 mg/day), cyclophosphamide (500 mg), and hydroxychloroquine (200 mg/day). The patient demonstrated gradual improvement in respiratory and congestive symptoms, with resolution of the pleural effusion. Although serum creatinine levels remain elevated, renal function has improved in the recent follow-up.

## Discussion

SLE is a chronic autoimmune disease usually diagnosed between the second and fifth decades of life. The 2019 EULAR/ACR criteria serve as the cornerstone for diagnosis, encompassing clinical and immunological domains. The female-to-male ratio in SLE varies significantly by age of onset. While it is markedly high (50:1) in patients aged 22-28, it drops to approximately 1.1:1 in late-onset disease [6]. Biologically, this narrowed sex gap is primarily attributed to hormonal changes, specifically the decline in estrogen levels in post-menopausal women and immunosenescence [6]. However, these data vary across studies depending on factors such as the criteria used to define late-onset SLE and the sociodemographic characteristics of each cohort. Observed geographic variability in late-onset SLE, particularly regarding sex ratios, likely stems from differences in study design and regional demographics. While some cohorts, such as those in Spain, report a marked increase in male prevalence, extensive studies in the United States and Latin America have found no significant difference compared to early-onset cases. These discrepancies often reflect recruitment biases [6].

The literature indicates that manifestations, such as malar rash, photosensitivity, alopecia, and renal involvement, as well as immunological markers, such as hypocomplementemia, anti-double-stranded DNA (anti-dsDNA), anti-Ro/SSA, anti-La/SSB antibodies, and anti-RNP, are generally less common in late-onset SLE [7]. This distinct clinical profile poses a significant diagnostic challenge. The absence of classic features, combined with the nonspecific nature of symptoms, often leads to misattribution to common geriatric comorbidities, resulting in diagnostic delays. Conversely, pulmonary involvement and serositis, specifically pleuritis and pericarditis, are more frequent in late-onset compared to early-onset cases [8]. Our case presents a distinct clinical profile. Serologically, the patient was consistent with late-onset SLE, with classical patterns, and had negative anti-dsDNA antibodies. However, the disease course was unusually aggressive for this demographic. It was characterized by severe hypocomplementemia and biopsy-proven class IV lupus nephritis, which are manifestations typically associated with early-onset disease.

The treatment approach for late-onset SLE generally emphasizes conservative management. This often includes the use of low-dose nonsteroidal anti-inflammatory drugs (NSAIDs), hydroxychloroquine, or glucocorticoids, as patients typically experience less disease activity, which may lessen the need for immunosuppressive therapy. 

In cases of severe disease activity, immunosuppressive therapy may be necessary. However, it is essential to conduct a thorough assessment of the patient's functional status, including physical and cognitive abilities, comorbid conditions, psychosocial and economic factors, and disease activity. Patients with late-onset SLE face an increased risk of iatrogenic harm, particularly infections, due to immunosenescence and age-related physiological decline.

**Figure 3:**
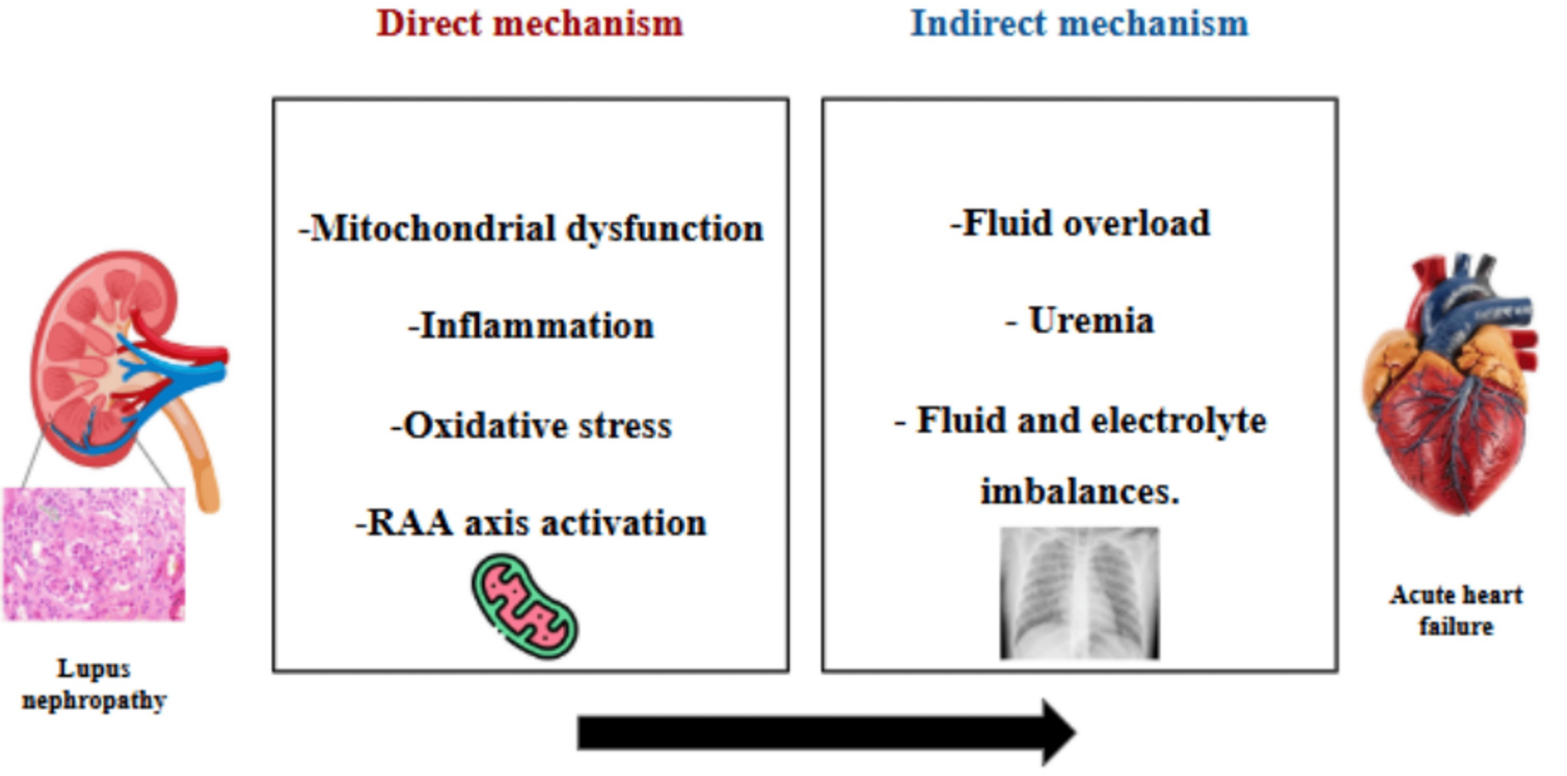
Direct and indirect mechanisms of type 3 cardiorenal syndrome in lupus nephropathy RAA: renin-angiotensin-aldosterone system Figure Source: Liu et al. [10]; reproduced under the Creative Commons Attribution License (CC BY 4.0)

CRS encompasses a spectrum of disorders involving concurrent renal and cardiac dysfunction, classified into five types [9]. Type 3 CRS is defined as acute kidney injury precipitating acute cardiac dysfunction. Although the precise mechanisms involve mitochondrial dysfunction, inflammation, and activation of the renin-angiotensin-aldosterone system (RAA) axis, indirect effects, such as fluid overload, are central (Figure 3). In our patient, the diagnosis of type 3 CRS is supported by the temporal sequence, in which severe lupus nephritis precipitated volume overload. However, we acknowledge a multifactorial pathophysiology: the massive pleural and pericardial effusions likely resulted from a synergistic interplay between renal-mediated fluid retention and SLE-associated inflammatory serositis [10].

Furthermore, regarding the cardiac dysfunction, although the echocardiogram reported a reduced LVEF (39%), this was interpreted primarily as a consequence of extrinsic hemodynamic constraint rather than intrinsic myocardial damage. The presence of a massive effusion with the "swinging heart" phenomenon and signs of pre-tamponade suggests that the ventricular hypokinesis was primarily mechanical, rather than a primary lupus myocarditis. However, an endocardial biopsy was not performed to confirm this hypothesis.

Distinguishing between type 5 CRS (systemic injury) and type 3 CRS (reno-cardiac) is challenging in SLE. However, in our patient, the clinical evidence strongly supports a type 3 classification [11]. While SLE was the underlying etiology, the acute cardiac decompensation was primarily driven by renal-mediated mechanisms rather than by direct myocardial inflammation [12]. Specifically, the severe hypoalbuminemia from lupus nephritis led to reduced oncotic pressure, precipitating the massive pericardial effusion and systemic volume overload [13]. Consequently, the cardiac dysfunction was a secondary mechanical and hemodynamic response to the acute kidney injury (transudative effusion), consistent with the pathophysiology of type 3 CRS rather than a simultaneous, independent involvement of both organs [14].

## Conclusions

Late-onset SLE is traditionally characterized by an insidious onset, nonspecific clinical manifestations, and a relatively benign course with lower organ involvement compared to early-onset disease, as described in recent regional cohorts. However, our case represents a distinct clinical outlier within this spectrum, illustrating a severe and diagnostically challenging debut of late-onset SLE manifesting as cardiorenal syndrome. While the patient's serological profile was consistent with late-onset disease, the presence of class IV lupus nephritis and massive pericardial effusion denotes an unusually aggressive clinical phenotype for a septuagenarian.

The key diagnostic pitfall highlighted by this case is the potential to overlook fulminant autoimmunity in elderly patients, often misattributing these findings to common age-related comorbidities. The absence of anti-dsDNA antibodies should not preclude the suspicion of lupus nephritis in the elderly, where definitive diagnosis relies on renal biopsy to guide life-saving interventions. Therapeutic decisions must always be based on a rigorous risk-benefit analysis, carefully weighing the potential complications of aggressive immunosuppression against the severity of disease activity.
